# Δ^9^-Tetrahydrocannabinol Differentially Alters Cannabidiol Efficacy in Recovery of Phonology and Syntax Following Damage to a Songbird Cortical-Like Brain Region

**DOI:** 10.1089/can.2022.0073

**Published:** 2023-10-09

**Authors:** Rachel M. Hodges, Katherine J. Chase, Mark A. Tripson, Sharon Bingham, Marie Woolley-Roberts, Geoffrey W. Guy, Ken Soderstrom

**Affiliations:** ^1^ Department of Pharmacology and Toxicology, ECU Brody School of Medicine, Greenville, North Carolina, USA.; ^2^ GW Research Ltd., Cambridge, United Kingdom.

**Keywords:** cannabinoid, vocal learning, animal model, CNS lesion

## Abstract

**Introduction:**

There are few vocal learning animals that are suitable for laboratory study, and so songbirds have unique utility for evaluating drug effects on behavior learned during a critical period of development. We previously found that purified botanically-derived cannabidiol (CBD, ≥98%) mitigates effects of partial ablation of zebra finch HVC, a pre-vocal motor cortical region. Here we expand prior work to determine ability of the euphorigenic cannabis constituent, Δ^9^-tetrahydrocannabinol (THC) to modulate CBD efficacy. Evidence suggests relative abundance of phytocannabinoids within cannabis extracts is an important determinant of activity, with CBD:THC of particular significance. As CBD-enriched extracts have become increasingly available both by prescription and over the counter, differential efficacy associated with distinct phytocannabinoid combinations and relative CBD:THC amounts is of increasing concern.

**Methods and Results:**

To evaluate THC modulation of CBD efficacy in mitigating the effects of partial ablation of zebra finch HVC, we have tested 3 mg/kg of purified botanically derived CBD (≥98%) containing 0.02, 0.08, 1, 3 and 5% THC. Results demonstrate differential efficacy on phonology and syntax, consistent with complex, hormetic dose-responses. On phonology, CBD with the lowest THC content (3% CBD + 0.02% THC) improved recovery while that with the highest THC content (3% CBD+5% THC) slowed it. In terms of syntax, all THC concentrations improved recovery time with the higher 3 mg/kg+3% THC being distinctly effective in returning behavior to pre-injury levels, and the highest 3 mg/kg CBD+5% THC for reducing the acute magnitude of syntax disruption. Differential phonology and syntax effects likely involve distinct neural circuits that control vocal learning and production. Understanding these systems-level effects will inform mechanisms underlying both phytocannabinoid action, and learning-dependent vocal recovery.

**Conclusions:**

Overall, we have found that efficacy of purified botanically derived CBD (≥98%) to influence vocal recovery varies with THC content in complex ways. This adds to evidence of differential efficacy with phytocannabinoid combinations and ratios thereof and underscores the importance of careful control over cannabis preparations used therapeutically.

## Introduction

Therapeutic use of natural products is complicated by variable abundance of active constituents, frequently with synergistic and/or antagonistic efficacies.^1^ These problems result in difficulty controlling dosages.^2^ The long history of cannabis use is accompanied by conflicting reports of variable efficacy and complex hormetic dose–response relationships.^3^ Such complex efficacy may involve nonstandardized preparations of myriad chemovars that produce varying levels of dozens of diverse bioactive molecules, including cannabinoids, flavones, terpenes, and others.^4,5^ Many of these cannabis-derived compounds interact with and modify the activity of multiple cellular targets, not all of which are yet well characterized.^5,6^

Accumulating evidence supports an entourage effect wherein distinct efficacy is produced by distinct combinations of active constituents present within different cannabis preparations.^7^ Recent expanded use of chemovars that produce high levels of the non-euphorigenic phytocannabinoid CBD has followed legalization of industrial hemp in the United States.^8^ Resulting CBD-enriched preparations have become increasingly available over the counter.^8^ Their unregulated nature illustrates the importance of understanding how CBD efficacy is modulated by other constituents, particularly THC that influences cognitive and perceptual processes.

We have previously used a songbird, the zebra finch, to investigate the drug effects on vocal learning.^9^ Using these animals in prior experiments, we found that purified botanically derived 10 mg/kg CBD (≥98%) improves learning-dependent recovery of vocal behavior following damage to HVC (used as a proper name), a brain region that functions as vocal premotor cortex.^10^ Subtle differences in effects of treatments prepared from different extracts led us to hypothesize that trace co-isolates modulate CBD efficacy, with cognitive and perceptually relevant THC being of particular concern.^11^ Based on prior dose–response experiments, we sought to establish an intermediately effective CBD dosage, settling upon purified botanically derived 3 mg/kg (≥98%). Our hypothesis was that partially effective CBD treatments will allow detection of either synergistic or antagonistic effects that varying THC content may have on measures of vocal recovery.

## Materials and Methods

### Materials

Unless otherwise indicated, all materials and reagents were purchased from Sigma or Fisher. Two lots of purified botanically derived CBD (≥98%) with differing trace amounts of THC (0.02% and 0.08%) and one lot of THC (96.2%) were provided by GW Research Ltd. (Cambridge, United Kingdom). All CBD treatments were prepared from material containing 0.02% THC, except for 3 mg/kg CBD + 0.08% THC prepared from the separate batch. Note that botanically derived CBD necessarily contains trace THC, precluding preparation of “CBD only” controls. We have adopted the convention to specify dosage of the principal constituent (in our case CBD) in mg/kg and amounts of less abundant co-isolates as percentages of the principal (i.e., THC as % CBD) used here-on-in. We have found this convention more efficient and less confusing than references to multiple dosages. Treatments were given in 50 μL intramuscularly into pectoralis as described previously^10^ and detailed in the Supplementary Data.

### Animals, microlesions, and audio recording

Animals, drug preparations, surgeries, audio recording, and analyses of vocal behavior were performed as described in detail previously^10^ and in the Supplementary Data. Animals were used according to protocols approved by ECUs Animal Care and Use Committee that is accredited by AAALAC International. Note that only male zebra finches learn to produce song, and so a limitation of our model is that effects on female vocal behavior cannot be investigated.

### Experimental design

**FIG. 1.**
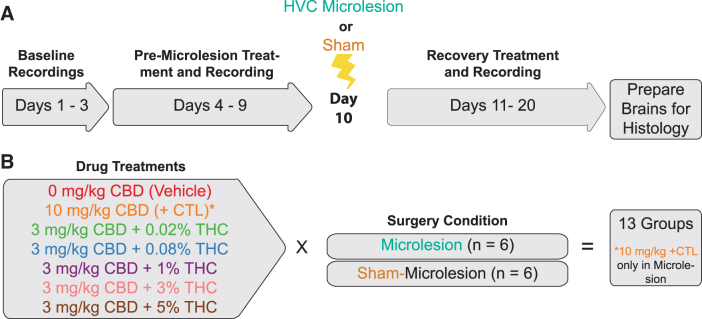
Study design. **(A)** Adult male subjects were recorded for 3 baseline days without other manipulation. Once daily treatments began on day 4. HVC (used as a proper name) microlesion or sham procedures were performed on day 10. Daily treatments and recordings continued to day 20. After day 20 recordings, animals were euthanized and brains prepared for histology. **(B)** Vehicle (2:1:17, ETOH:Alkamuls:PBS) and 10 mg/kg CBD + 0.02% THC positive controls were used along with 3 mg/kg CBD treatment groups with 0.02%, 0.08%, 1%, 3%, and 5% THC. Groups of *n*=6 animals each were used×13 groups for 78 animals total. PBS, phosphate-buffered saline.

The treatment plan is summarized in Figure 1. The same 20-day schema employed previously was reemployed here (detailed procedures are described in the Supplementary Data).^10^ Briefly, during the first 3 days, no treatments were given, and recordings were used to generate baseline measures to which later daily recordings were compared. After baseline recordings, once daily morning treatments began.

### Kullback–Leibler distance measures of phonology

Kullback–Leibler (KL) distance is a measure of acoustic divergence between song syllables produced during baseline and treatment day recordings, with greater values indicating greater divergence. KL distance measures were calculated from daily audio recordings as described previously (see the Supplementary Data for details).^10^

### Typical syllable transition measures of syntax

Syntax was measured from the frequency of typical syllable transitions that were calculated using the SongSeq software according to the published methods^12^ and as performed previously^10^ (see the Supplementary Data for details).

### Microglial densities

Adult male zebra finches (*n*=3 per group) were treated for 2 days pre- and post-microlesion with vehicle or 10 mg/kg CBD +0.02% THC. Two days post-microlesion, animals were transcardially perfused, brains dissected, and cryo-sectioned at 10 μm. Sections were blocked in phosphate-buffered saline/1% goat serum/0.01% Triton X-100 and stained with 1:1000 isolectin *GS*-IB4 (No. I21411; Thermo Fisher). This reagent interacts with vascular endothelium but most notably labels microglia.^13^ Total cells were determined by Hoechst counterstaining (1:10,000, H3570; Thermo Fisher Scientific). Microglia densities were calculated and expressed as a fraction of the total cell number. Confocal images were taken at 20×magnification using DAPI and GFP lasers and images analyzed with the FIJI software.

### Statistical analyses

Lesion extents were assessed using one-way analysis of variance (ANOVA). Microglia density differences were determined via two-tailed *t*-test. Differences in phonology and syntax were assessed using a mixed-effects modeling approach as described previously and detailed in the Supplementary Data.^10^

## Results

### Lesion extent

**FIG. 2.**
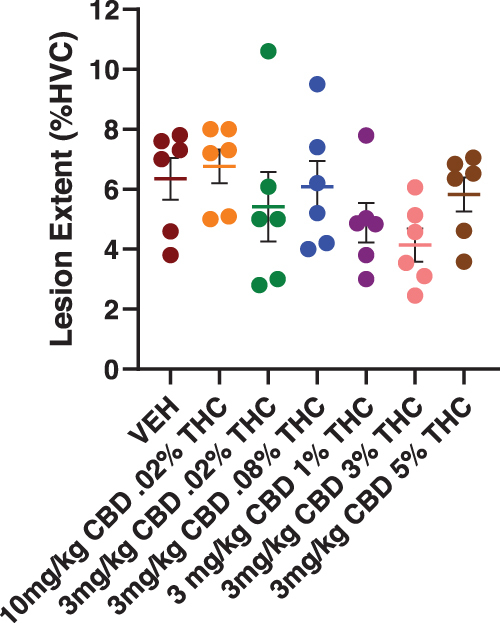
Lesion extent was measured using the ImagePro software to trace both HVC and infarcts, if present. Tissues were Nissl stained, and all HVC-containing sections were imaged. Lesion extent=infarct area/HVC area×100%. The mean lesion extent was 5.6%±0.34% of HVC and did not differ across treatment groups [one-way ANOVA, *F*(6,35)=1.44, *p*=0.23]. ANOVA, analysis of variance.

Overall mean lesion extent was 5.6%±0.34% (mean±standard error of the mean [SEM]) (Fig. 2). The mean %HVC damaged did not significantly vary across treatments [one-way ANOVA, *F*(6,35)=1.44, *p*=0.230]. Lesion extents produced in the present study were lower than previously observed (8.6%±0.74%).^10^ Improved equipment and experience likely increased procedure efficiency, reducing damage. Reduced lesion extents did not notably alter magnitudes of vocal disruption.

### Mixed models fit to phonology and syllable sequence data

Mixed model fit of vocal behavior measures to explanatory variables was optimized as described previously and detailed in the Supplementary Data.^10^ For measures of both phonology and syntax, addition of surgery condition, treatment group, and experiment day sequentially improved the fit of models to explanatory variables, demonstrating significant effects of each variable. Likelihood ratios and resulting χ^2^ statistics are presented in detail in the Supplementary Data.

### Recovery of phonology

**FIG. 3.**
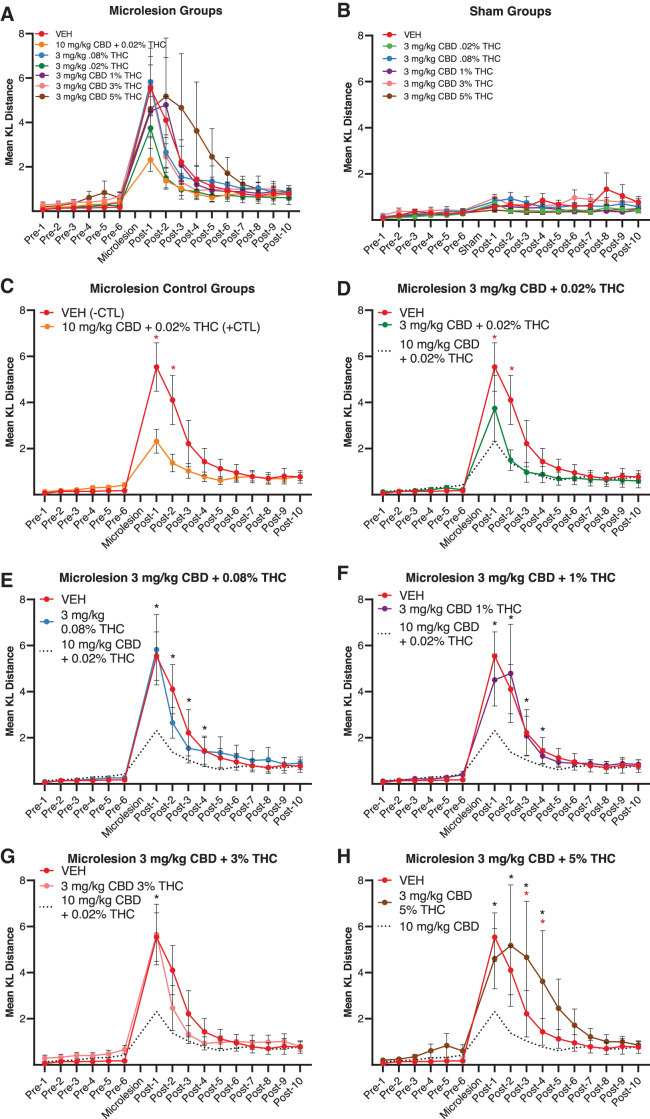
KL distance measures of phonology by surgery and drug treatment group. Higher KL distance measures reflect greater pre- and post-microlesion acoustic divergence. **(A, B)** Summarize KL distance measures in microlesioned and sham-microlesioned groups, respectively. **(B)** Following mixed models analysis, no significant differences were observed across treatment groups in sham-microlesion animals. **(C)** Ten milligrams per kilogram CBD + 0.02% THC was found previously to reduce KL distances and was used here as a positive control. Significant differences from vehicle groups on post-microlesion days 1 and 2 are indicated by red asterisks. **(D)** Three milligrams per kilogram CBD + 0.02% THC lowered KL distance measures on the first 2 recovery days relative to vehicle (red asterisks). This lowest THC treatment appeared less effective than 10 mg/kg CBD + 0.02 % THC (dashed line), although the difference was not significant according to Bonferroni-corrected *post hoc* tests. **(E–H)** Three milligrams per kilogram CBD + 0.08%, 1%, 3%, and 5% THC were all less effective than the 10 mg/kg CBD + 0.02% THC positive control (black asterisks). **(H)** Three milligrams per kilogram CBD + the highest 5% THC concentration significantly slowed recovery relative to vehicle (red asterisks). Shown are mean values±SEM. KL, Kullback–Leibler; SEM, standard error of the mean.

KL distance measures of phonology are summarized in Figure 3A and B, respectively. As detailed in Table 1 and the Supplementary Data, mixed models analyses confirmed significant phonology differences between microlesion and sham surgery groups. Despite a main effect of surgery condition, no significant *post hoc* treatment group differences within sham-lesioned animals were noted (Fig. 3B).

**Table 1.** Kullback–Leibler Distance Measures of Phonology, *Post Hoc* Comparisons to the Vehicle Group Following Mixed Models Analysis

	3 mg/kg CBD +					10 mg/kg CBD + 0.02% THC positive CTL
	0.02% THC	0.08% THC	1% THC	3% THC	5% THC	
Post-1						
Difference	**1.88**	0.47	0.89	0.36	0.38	**3.29**
*p*	**0.045**	1.000	1.000	1.000	1.000	**<0.001**
Post-2						
Difference	**2.61**	1.21	0.57	1.46	0.94	**2.73**
*p*	**<0.001**	1.00	1.00	1.00	1.00	**<0.001**
Post-3						
Difference	1.25	0.67	0.13	0.9	2.45	1.19
*p*	1.000	1.000	1.000	1.000	0.002	0.656
Post-4						
Difference	0.56	0.02	0.23	0.5	**2.19**	0.66
*p*	1.000	1.000	1.000	1.000	**0.002**	1.000
Post-5						
Difference	0.43	0.22	0.19	0.15	1.32	0.52
*p*	1.000	1.000	1.000	1.000	0.348	1.000
Post-6						
Difference	0.24	0.24	0.06	0.06	0.76	0.21
*p*	1.000	1.000	1.000	1.000	1.000	1.000
Post-7						
Difference	0.12	0.23	0.1	0.2	0.43	0.01
*p*	1.000	1.000	1.000	1.000	1.000	1.000
Post-8						
Difference	0.07	0.34	0.09	0.28	0.3	0.02
*p*	1.000	1.000	1.000	1.000	1.000	1.000
Post-9						
Difference	0.18	0.05	0.08	0.21	0.19	0.11
*p*	1.000	1.000	1.000	1.000	1.000	1.000
Post-10						
Difference	0.17	0.13	0.08	0.03	0.09	0.02
*p*	1.000	1.000	1.000	1.000	1.000	1.000

Bold font indicates Bonferroni-corrected significant differences.

CTL, control.

We previously found that 10 mg/kg CBD +0.02% THC was effective in reducing both magnitude of phonology deficits and time to recovery. Using this treatment in the present study as a positive control, results confirmed efficacy to reduce phonology disruptions (Fig. 3C, and black dashed lines in Fig. 3D–H). Ten milligrams per kilogram of CBD +0.02% THC significantly reduced KL distance measures on post-microlesion days 1 and 2 (Fig. 3C). Phonology statistics (mean differences, SEM, and *p*-values) are summarized in Table 1.

A goal of the present study was to establish a partially effective CBD dosage capable of being either potentiated or diminished in efficacy through addition of various concentrations of THC. We found that 3 mg/kg CBD +0.02% THC significantly improved phonology and appeared less effective than 10 mg/kg CBD +0.02% THC (although *post hoc* differences were not significant on any post-microlesion day). Like 10 mg/kg CBD +0.02% THC, 3 mg/kg CBD +0.02% THC significantly lowered KL distance measures on post-microlesion days 1 and 2 (Fig. 3D).

Three milligrams per kilogram of CBD combined with 0.08%, 1, 3%, and 5% THC groups had significantly higher KL distance measures on recovery day 1 than 10 mg/kg CBD +0.02% THC (Fig. 3E–H, black asterisks, red=comparisons to vehicle). KL distances were significantly lower in 10 mg/kg CBD +0.02% THC-treated animals than in groups that received 3 mg/kg +0.08%, 3%, and 5% THC on recovery day 2, and less than the 5% THC group on recovery days 3–5. Lack of a similar significant difference between 10 mg/kg CBD +0.02% THC and 3 mg/kg CBD +0.02% THC is evidence of greater CBD efficacy in the presence of the lower THC concentration.

It is of particular note that in the case of 3 mg/kg CBD +5% THC, recovery took significantly longer than in vehicle controls, demonstrating an effect of the highest THC concentration to oppose CBD-improved phonology (note rightward shift of KL distance curve, Fig. 3H). This demonstrates that differences in THC content may lead to significantly altered effectiveness of CBD extracts.

### Recovery of syntax

**FIG. 4.**
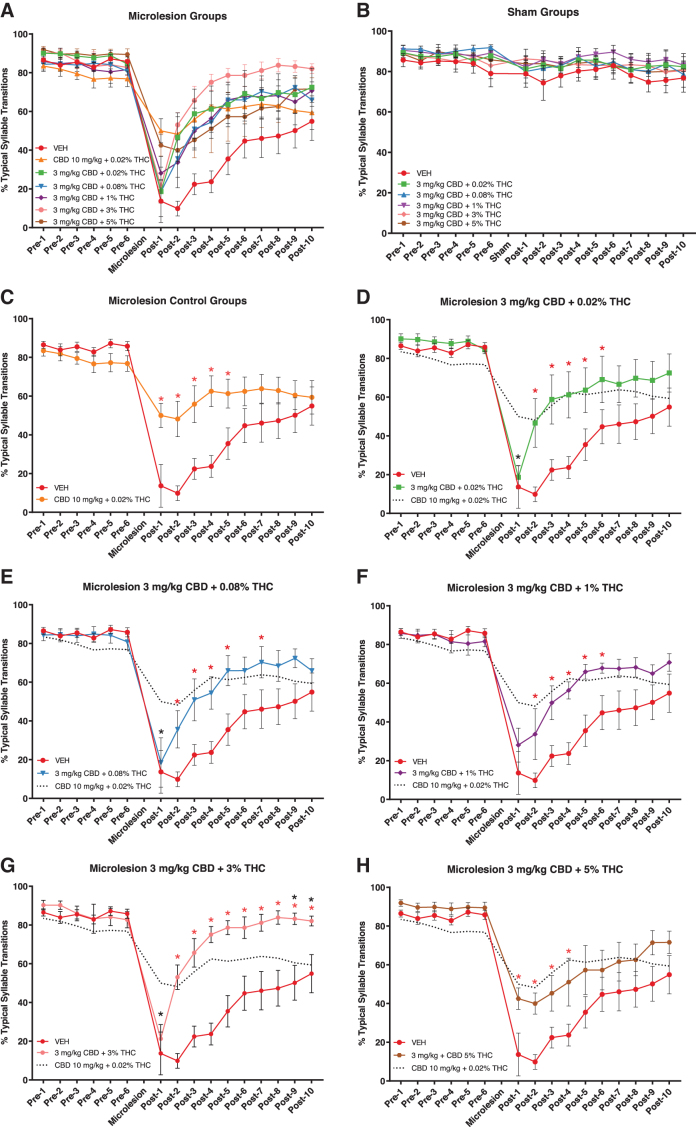
Syntax quality measured through percent typical syllable transitions. Higher measures indicate a more stereotyped song. **(A, B)** Summarize typical transition percentages in microlesioned and sham-microlesioned groups, respectively. **(B)** Following mixed models analysis, no significant treatment group differences were observed in sham-microlesioned animals. **(C)** The 10 mg/kg CBD + 0.02% THC positive control (formulated from material with 0.02% THC) reduced the magnitude of microlesion effects on syntax and reduced recovery time. **(D)** Three milligrams per kilogram CBD + 0.02% THC was less effective than 10 mg/kg CBD + 0.02% THC (dashed line) in reducing the magnitude of syntax disruption (black asterisk) but had similar efficacy in reducing recovery time relative to vehicle controls (red asterisks). **(E–H)** Similar patterns of efficacy were observed in groups receiving 3 mg/kg CBD + 0.08%, 1%, 3%, and 5% THC with exceptions. **(G)** Syntax in the 3% THC group was more fully restored to pre-microlesion levels than in the 10 mg/kg + 0.02% THC positive control (black asterisks). **(H)** The magnitude of syntax disruptions was reduced by the 5% THC treatment to levels similar to that of the 10 mg/kg CBD + 0.02% THC positive control. Shown are mean values±SEM.

Typical syllable transition measures of syntax are summarized for microlesion and sham-microlesion groups in Figure 4A and B, respectively. As described above, fitting percent typical transition data to mixed models demonstrated main effects of each fixed factor: drug treatment, surgery condition, and experiment day. *Post hoc* tests did not reveal significant drug treatment group differences in sham-microlesioned animals (Fig. 4B).

*Post hoc* statistics for the microlesion group drug treatment differences from vehicle control (mean differences, SEM, and *p*-values) are presented in Table 2 and are summarized in Figure 4C–H. As expected from prior work, 10 mg/kg CBD +0.02% THC significantly increased percent typical syllable transitions relative to vehicle on post-microlesion days 1–4 (Fig. 4C). Interestingly, the dosage group receiving the highest THC concentration (3 mg/kg CBD +5% THC) had a similar pattern of efficacy with significantly higher typical transitions on the first 4 recovery days (Fig. 4H). Higher percent typical transitions on the first recovery day in 10 mg/kg CBD +0.02% THC and 3 mg/kg CBD +5% THC groups suggest that both treatments were acutely effective in reducing the magnitude of microlesion effects on syntax. No other treatment was associated with increased typical syllable transitions on the first recovery day, suggesting lack of acute efficacy to reduce the magnitude of syntax disruptions.

**Table 2.** Percent Typical Syllable Transition Measures of Syntax, *Post Hoc* Comparisons to Vehicle Group Following Mixed Models Analysis

	3 mg/kg CBD +					10 mg/kg CBD +0.02% THC positive CTL
	0.02% THC	0.08% THC	1% THC	3% THC	5% THC	
Post-1						
Difference	11.4	5.0	18.8	7.9	**27.7**	**41.7**
*p*	1.000	1.000	0.459	1.000	**0.027**	**<0.001**
Post-2						
Difference	**36.8**	**25.8**	**24.6**	**40**	**27.6**	**38.3**
*p*	**<0.001**	**0.01**	**0.025**	**<0.001**	**0.006**	**<0.001**
Post-3						
Difference	**36.4**	**28.5**	**27.5**	**43.2**	**22.8**	**33.4**
*p*	**<0.001**	**0.003**	**0.005**	**<0.001**	**0.042**	**<0.001**
Post-4						
Difference	**37.5**	**30.7**	**32.6**	**51.4**	**27.4**	**38.8**
*p*	**<0.001**	**0.001**	**<0.001**	**<0.001**	**0.005**	**<0.001**
Post-5						
Difference	**28.2**	**30.5**	**30.5**	**43.2**	21.8	**25.9**
*p*	**0.003**	**0.001**	**0.001**	**<0.001**	0.066	**0.010**
Post-6						
Difference	**24.4**	21.2	**23.1**	**33.9**	12.6	17.7
*p*	**0.021**	0.084	**0.038**	**<0.001**	1.000	0.339
Post-7						
Difference	20.6	**24.2**	21.5	**35.1**	15.5	17.7
*p*	0.111	**0.023**	0.075	**<0.001**	0.726	0.340
Post-8						
Difference	22.4	21.1	20.9	**36.5**	15.2	15.6
*p*	0.051	0.091	0.099	**<0.001**	0.803	0.724
Post-9						
Difference	18.6	22.2	14.9	**33.1**	21.3	10.4
*p*	0.248	0.056	0.901	**<0.001**	0.084	1.000
Post-10						
Difference	17.6	11	15.8	**27.2**	16.7	4.5
*p*	0.353	1.000	0.659	**0.005**	0.486	1.000

Bold font indicates Bonferroni-corrected significant differences.

The other treatments did significantly reduce time to recovery. In the case of 3 mg/kg CBD + 0.02% THC, typical transitions were significantly increased by the second recovery day and remained higher than control through post-microlesion day 6 (Fig. 4D). Similar responses were observed in animals treated with 3 mg/kg CBD +0.08% and 1% THC (Fig. 4E, F). Although 3 mg/kg CBD + 3% THC did not appear to reduce the magnitude of microlesion impact (Fig. 4G), typical transitions did not differ from vehicle the day after lesions but did differ from the positive control. The 3% THC-containing treatment did notably improve recovery time and, in addition, more completely restored syntax to presurgery levels than other treatments: for example, compared with 10 mg/kg + 0.02% THC, 3 mg/kg CBD + 3% THC produced significantly higher typical syllable transitions on the last 2 days of the experiment.

### Microglia density

**FIG. 5.**
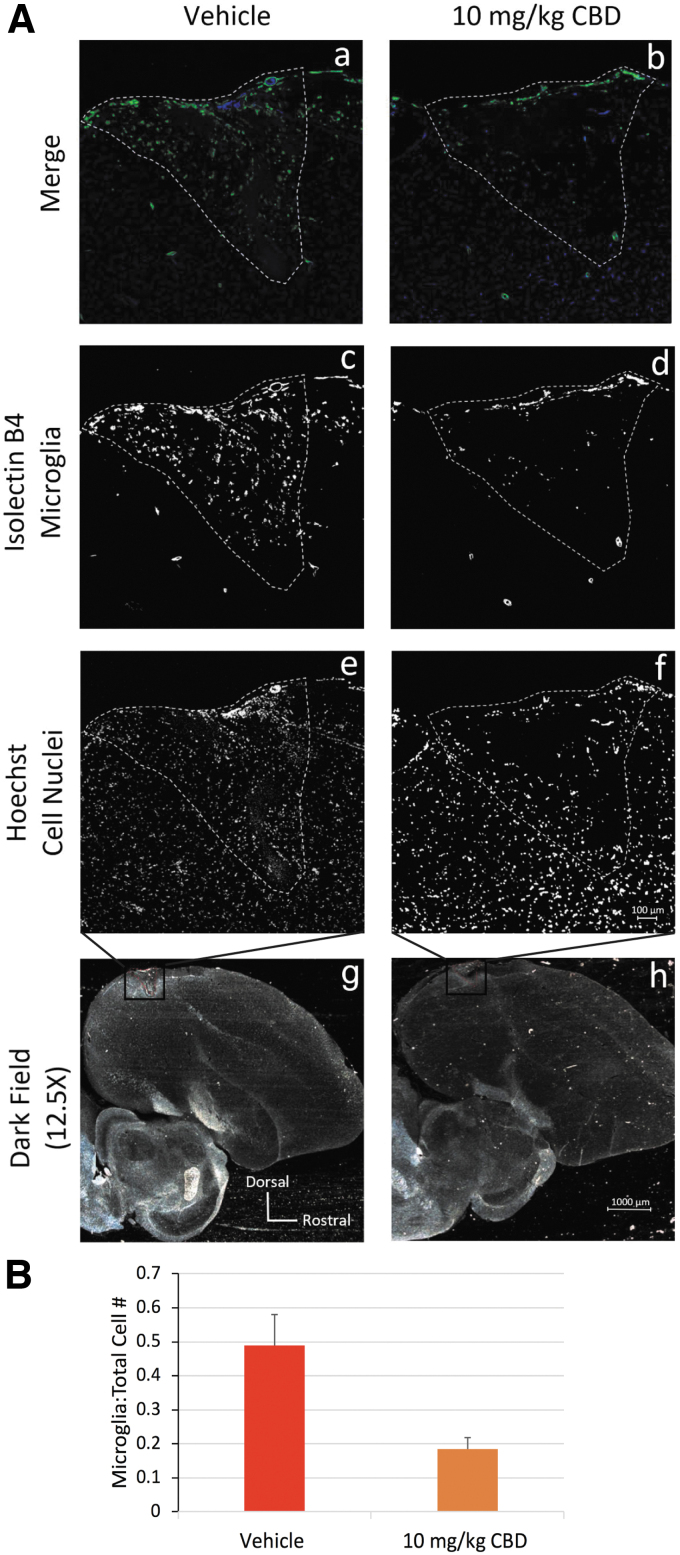
IF imaging and quantification of the microglial marker isolectin B4,^13^ *n*=3 for each treatment. **(A.a–A.f)** IF imaging at 200×sections of representative HVC microlesioned zebra finch brains treated with either the vehicle or 10 mg/kg CBD +0.02% THC with isolectin B4 (green) and DAPI (blue). White outline describes microlesioned areas. **(A.g, A.h)** Darkfield images at 12.5×of parasagittal sections. Red-dotted line outlines HVC. **(A.c–A.h**) Black box. **(B)** Quantification of isolectin B4-positive cells normalized to total cells in the lesioned area. Two-tailed *t*-test *p*<0.03. Error bars represent 1 standard deviation. IF, immunofluorescent.

To begin appreciating mechanisms responsible for CBD efficacy in this system, we studied microglial densities well established in other systems to be part of the drug's action.^14^ Figure 5A micrographs show differential patterns of lesion site staining with both the microglial marker isolectin B4 and Hoechst staining of cell nuclei in tissue from vehicle-treated (Fig. 5A.a, A.c, A.e, A.g) and 10 mg/kg CBD + 0.02% THC-treated animals (Fig. 5A.b, A.d, A.f, A.h, marker=100 μm). Vehicle treatments resulted in clusters of isolectin B4-stained microglia (Fig. 5A.c) at the lesion site. Ten milligrams per kilogram of CBD + 0.02% THC (Fig. 5A.d) shows less clustered dispersed microglia. Lower power darkfield images in Figure 5A.g and A.h illustrate orientation of lesion sites (outlined in red, marker=1000 μm). Cell counts indicate that 10 mg/kg CBD + 0.02% THC significantly reduced microglia densities (Fig. 5B, total microglia normalized to total cells differed by 0.30±0.12, *p*=0.030).

## Discussion

### Intermediate CBD efficacy

The HVC microlesion pre-clinical model was originally established through a dose–response experiment that investigated effects of 10-fold increasing CBD dosages on vocal recovery.^10^ Results indicated approximately threshold responses following 1 mg/kg treatments and full efficacy at 10 mg/kg CBD + 0.02% THC. In an effort to produce an intermediate level of response that is subject to both positive and negative modulation by additional treatments (e.g., increasing THC concentrations), we employed a half-log dosage of 3 mg/kg CBD + 0.02% THC as an intermediate dose between the prior 1 mg/kg threshold and 10 mg/kg + 0.02% THC full efficacy dosages. In terms of phonology, although this 3 mg/kg CBD + 0.02% THC treatment appeared slightly less effective than the 10 mg/kg CBD +0.02% THC positive control, the difference was not statistically significant, and the lower dosage was equally effective in reducing KL distance measures on the first 2 recovery days (Fig. 3D).

In contrast, on measures of syntax, 3 mg/kg CBD +0.02% THC was less effective than 10 mg/kg CBD +0.02% THC to reduce the acute magnitude of disruption immediately following microlesions, and no more effective than vehicle (Fig. 3D, note that this treatment did hasten phonology recovery relative to vehicle). This differential responsiveness may be seen as inconsistent with the now well-documented “entourage effect” wherein combinations of phytocannabinoids, even in trace amounts, synergize to produce greater efficacy.^15^ It may be the case that both synergistic and antagonistic entourage effects are possible depending on relative phytochemical abundance and expression of the cellular macromolecules they target.

Our results demonstrate a challenge in establishing a CBD treatment with clear intermediate efficacy on vocal recovery. This difficulty is likely due to a combination of modest response levels separating threshold and the fully effective 10 mg/kg CBD + 0.02% THC dosages, subject variability inherent to all behavioral experiments and well-established complex phytocannabinoid effects.^3^ Clear intermediate efficacy was also difficult to produce due to interesting differential responsiveness of phonology and syntax measures. These sensitivity differences were more evident at the higher THC concentrations as detailed below.

### THC modulation of CBD efficacy

#### Phonology

KL distance phonology measures were modestly improved by 3 mg/kg CBD + 0.02% THC. Notably, improvements were not observed in animals treated with 3 mg/kg CBD containing slightly higher 0.08% THC (compare Fig. 3D, E). It may be important that the 0.02% and 0.08% THC-containing CBD extracts were from independent lots and so likely differed in content of other trace phytochemicals. Our results add to accumulating evidence that even small differences in trace amounts of phytocannabinoids may significantly influence extract activity.^16,17^

In contrast to 3 mg/kg CBD + 0.02% THC, the higher THC concentration treatments (3 mg/kg CBD +0.08%, 1%, 3%, and 5% THC) were all less effective than the 10 mg/kg CBD + 0.02% THC positive control to reduce KL distance measures. Notably, the highest 3 mg/kg CBD +5% THC dosage was distinguished also by slower restoration of phonology than by the vehicle-treated group. This demonstrates an effect of higher THC concentrations to oppose CBD-promoted recovery of phonology-relevant spectral features. Transforming the 5% THC treatment to dosing units results in 0.15 mg/kg, approximating threshold THC dosages for mouse locomotor effects.^18^ This suggests that the 5% THC-containing treatment delayed phonology recovery via effects upon motor function, a possibility more likely given that microlesion recovery requires auditory-dependent sensorimotor learning.^19^

#### Syntax

In terms of reducing time to recovery of syntax, each of the 3 mg/kg CBD dosages (+ 0.02%, 0.08%, 1%, 3%, and 5% THC) was similar in efficacy to the 10 mg/kg CBD + 0.02% THC positive control. In addition to decreasing recovery time relative to vehicle controls, 3 mg/kg CBD + 5% THC also reduced the acute magnitude of syntax disruptions in a manner similar to the 10 mg/kg CBD + 0.02% THC positive control (Fig. 4H). This suggests a protective effect of the highest THC concentration that contrasts with its slowing of phonology recovery discussed above.

The second highest 3 mg/kg CBD + 3% THC dosage did not produce this acute reduction in magnitude of syntax disruption but did produce a novel effect to more fully restore percent typical transitions to pre-microlesion levels before the end of experiments. This was evident from typical syllable transition levels significantly higher than the 10 mg/kg CBD + 0.02% THC positive control on the last 2 recovery days (Fig. 4G, it may be important that, although there were not significant differences in lesion extent across treatment groups, animals receiving 3 mg/kg CBD + 3% THC did have the lowest mean percent HVC damaged).

### Differential efficacy on phonology and syntax

We previously found that 10 mg/kg CBD with 0.02% THC mitigates microlesion effects to disrupt phonology and syntax, and viewed these measures as indicative of a single comprehensive effect: to improve vocal recovery.^10^ The present results suggest that this interpretation was too limited, and phonology and syntax are subject to differential phytocannabinoid modulation.

**FIG. 6.**
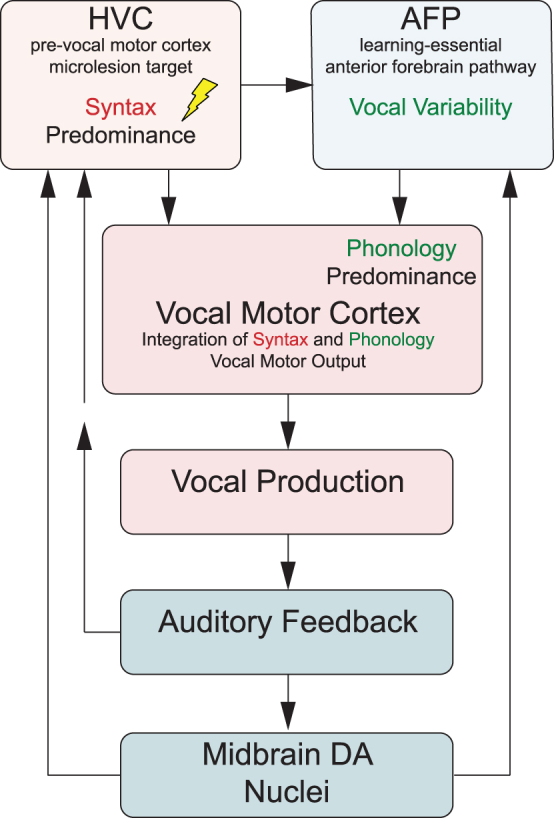
Two major song system pathways differentially contribute to vocal phonology and syntax. The premotor cortical-like microlesion target HVC projects to vocal motor cortex influencing temporal and syllable sequence features relevant to syntax.^29^ The learning-essential AFP also projects to vocal motor cortex and is predominantly responsible for promoting vocal variability, particularly of spectral features important to phonology.^30^ Note that vocal impairment following HVC microlesions requires an intact AFP input to vocal motor cortex, suggesting that its variability signal is normally balanced by HVC.^31^ Microlesion recovery depends on auditory feedback.^19^ This is consistent with song degradation following deafening^32^ that also depends on intact AFP.^33^ Sensorimotor learning refinement of song involves midbrain dopaminergic circuits that project to both AFP^34^ and HVC.^35^ AFP, anterior forebrain pathway.

Phonology and syntax are largely controlled by distinct, but converging, neural signaling pathways that interconnect brain regions important to vocal learning and motor production of song (Fig. 6). For example, the pre-vocal motor brain region we have targeted with microlesions, HVC, is most clearly involved in regulating the temporal structure of song.^20,21^ Thus, activity within HVC plays a prominent role in controlling syntax. The clear efficacy of CBD to reduce microglial densities within HVC microlesion sites (Fig. 5) suggests a potentially direct anti-inflammatory effect of CBD to stabilize HVC function and related syntax.

Control of phonological features of song syllables is more complex and related to combined influences of a learning-essential basal ganglia-thalamo-cortical circuit called the anterior forebrain pathway (AFP) (Fig. 6).^22^ This circuit sends excitatory projections to the vocal motor cortical-like region, robust nucleus of the arcopallium (RA).^23,24^ Notably, AFP output contributes variability to song structure^25,26^ that is integrated with timing-related input from HVC at the level of vocal motor cortical RA.^27,28^ The vocal variability produced by the AFP is essential to normal vocal learning that requires progressive sensorimotor refinement of vocalizations.^26^ Microlesion recovery depends on this AFP-dependent sensorimotor learning (because deafened zebra finches do not relearn song^19^) and so phonology improved by CBD must involve action within one or more AFP elements. Identifying these elements will be key to understanding how CBD-enriched extracts improve learning-dependent vocal recovery, and how THC modulates this efficacy.

## Conclusions

We have found that 3 mg/kg purified botanically derived CBD (≥98%) with a trace amount of THC (0.02%) effectively improves vocal recovery following damage to a pre-vocal motor cortical-like region and reduces impact on measures of phonology and syntax. Increasing THC content in the 3 mg/kg CBD treatments (from 0.02% to 0.08%, 1%, 3%, and 5%) generally slowed recovery of phonology features while conversely promoting recovery of motor-related syntax. These results demonstrate that even small differences in relative amounts of CBD and THC can result in modified efficacy. This underscores the importance of careful and consistent formulation of cannabis preparations.

## Supplementary Material

Supplemental data

## Abbreviations Used

AFP: anterior forebrain pathway
ANOVA: analysis of variance
CBD: cannabidiol
IF: immunofluorescent
KL: Kullback–Leibler
PBS: phosphate-buffered saline
RA: robust nucleus of the arcopallium
SEM: standard error of the mean
THC: Δ^9^-tetrahydrocannabinol
