# Supplementary material for: Δ9-Tetrahydrocannabinol Differentially Alters Cannabidiol Efficacy in Recovery of Phonology and Syntax Following Damage to a Songbird Cortical-Like Brain Region
Source: Cannabis Cannabinoid Res. 2023 Oct 9;8(5):790–801. doi: 10.1089/can.2022.0073 (PMC10589500; doi:10.1089/can.2022.0073)
Supplement: Supplemental data [file Supp_DataS1.docx]

# Supplemental introduction material

In the United States, industrial hemp is characterized by low, but variable, levels of the euphorigenic cannabinoid Δ^9^-tetrahydrocannabinol (THC, typically < 0.3%) while its CBD and other phytocannabinoid content remain unregulated^1^.

We found previously^2^ that purified botanically derived CBD (≥98%)-related improvement in phonology and syntax were observed following daily treatments with 10 and 100 mg/kg that produced similar levels of efficacy, whereas CBD at 1 mg/kg produced little effect. Similar efficacies of the higher, 10-fold differing dosages suggest a maximal level of response was achieved upon reaching the 10 mg/kg dosage.

One of our goals in the current project was to establish a CBD dosage that produces something less than full efficacy to “improve vocal recovery” that may either be augmented or antagonized. This led to our adoption of 3 mg/kg CBD that is approximately a half-log unit between the earlier determined threshold of 1 mg/kg and fully efficacious 10 mg/kg CBD dosages. We didn’t expect to find differential effects upon syntax and phonology. This, of course, was an interesting result and we try to make it a focus of the paper. But it has also complicated interpretation of the results, and we can no longer say that CBD simply “improves vocal recovery” as its efficacy clearly depends upon the amount of THC coextracted and remaining in the preparation.

# Supplemental methods details

## Drug treatments

Stocks of purified botanically derived CBD and THC, prepared as described before^2^, were stored in sterile 2 or 5 ml septum-capped vials at 4 °C. Low actinic amber vials were used to minimize photodegradation. Fresh stocks were prepared at least weekly. For injections, drug preparations were loaded into sterile 1 ml insulin syringes with 30 gauge needles. Just before lights-on, zebra finches in recording chambers were captured by hand and breast muscle injection sites exposed by matting feathers with a small volume of 70% ethanol delivered by squirt bottle. Injections of 50 μl were made into one of four quadrants of pectoralis, rotating daily to minimize potential damage caused by repeated treatments.

## Supplemental experimental design details

Six daily treatments were given prior to surgical procedures for two reasons: (1) to assess potential for CBD administration without other interventions to alter vocal behavior, and (2) in an effort to allow CBD, a lipophilic drug with large volume of distribution and elimination half-life, to approximate steady-state levels^3^. Surgeries were done on day 10 and treatments and recordings continued to follow recovery to day 20

## Supplemental zebra finch and audio recording environment details

Adult male zebra finches (>90 days of age) were raised in our breeding aviary and maintained at 25.6 °C on a 12/12 light/dark cycle. Zebra finches were individually housed in standard finch cages (23 x 28 x 43 cm) placed within recording chambers with *ad libitum* food and water. Chambers were fitted with individual microphones and lights. Zebra finches were visually isolated while continuously recorded. Sound Analysis Recorder software^4^ was used to record vocalizations and store in Waveform Audio File (WAV) format. Zebra finches were placed in recording chambers several days (3 to 5) prior to experiments. Zebra finches not producing at least 500 song bouts per day during the last three habituation days were excluded (about 10% of zebra finches are excluded due to infrequent singing). All zebra finch procedures were approved by the East Carolina University Animal Care and Use Committee. Note that females were not used in this study as female zebra finches do not produce song.

Group numbers were n=6 zebra finches per treatment (vehicle, 10 mg/kg CBD + 0.02% THC and 3 mg/kg CBD + 0.02, 0.08, 1, 3 and 5% THC, Fig 1) and surgery condition (microlesion and sham-microlesion). Note that the 10 mg/kg CBD + 0.02% THC positive control treatment was formulated from the same material as the 3 mg/kg CBD + 0.02% THC treatment group, and therefore also contained the same trace amount of 0.02% THC. As a sham control was previously done for the 10 mg/kg CBD dosage^2^ and this treatment group was only included as a positive control, the sham condition for this treatment was not repeated in the present study. Thus, the number of zebra finches used was six (group size) times seven (treatment groups) times two (surgery conditions) less the positive control sham equals 78 adult male zebra finches total. Animal use at East Carolina University (ECU) is accredited by AAALAC International and experiments described herein were done according to protocols approved by ECU’s Animal Care and Use Committee.

## Supplemental microlesion surgery details

Bilateral HVC microlesions were made following the procedure previously described^5^ except that zebra finches were administered the anti-inflammatory drug meloxicam (1 mg/kg) prior to procedures and were anesthetized with isoflurane. zebra finches were secured in a stereotaxic instrument (Stoelting’s mouse version modified with digital manipulator arms and a small bird adaptor) and the bifurcation at the midsagittal sinus was used as stereotaxic zero. Nixdorf-Bergweiler and Bischof’s stereotaxic atlas was used to guide electrode placement^6^. Small craniotomies were placed over HVC bilaterally. For approximately 6% destruction of HVC, four locations were targeted: 2.4 and 2.8 mm bilaterally from stereotaxic zero to a depth of 0.6 mm. Microlesions were made with 100 μA for 35s. Zebra finches recovered in a warm incubator and were returned to recording chambers. Sham-group animals were subjected to all of the steps described above (including anesthesia, craniotomies and suturing) except no current was passed through electrodes.

## Supplemental KL distance measures of phonology details

Zebra finches were recorded continuously over the entire 20-day experimental period. Recording files were input to Sound Analysis Pro 2011 software (SAP^4^) to segment song bouts into their separate syllable components (uttered sounds separated by silence). Segmentation was accomplished by thresholding based upon amplitude, entropy, syllable and syllable gap durations. Thresholding was optimized for each zebra finch, all other procedures were done objectively. Others have reported^7^ and we confirm that analyses of fewer than approximately 300 syllables is associated with underestimated phonology quality. Syllable numbers rarely reached 300 on microlesion days and so these days were excluded from analyses. Other days not meeting this criteria (typically due to power interruptions or automated operating system upgrades to computers controlling recording systems) were also excluded.

SAP characterizes individual syllables by their spectral structure through measures of acoustic features (e.g. syllable duration, amplitude, pitch, etc.) Acoustic feature measures were used to assess phonology via calculation of Kullback-Leibler (KL) distances using the methods developed by ^7,8^ that compare distances between 2D probability distributions of vocal acoustic features. Greater KL distance measures reflect increased phonological divergence across the vocalizations compared. We used acoustic measures from baseline recordings (days 1–3, Fig. 1B) as the “template” distribution, and recordings from following treatment days as individual “targets”. KL distances between template and target distributions were calculated using software we developed (KLFromRecordingDays^9^). Because baseline days were used as the template, KL distances for this period = 0, and higher values observed on following days represent phonological divergence from baseline measures. Significant KL distance measure outliers, detected by the method described by^10^, were excluded from statistical analysis.

## Supplemental typical syllable transition measures of syntax details

Syntax was measured from the frequency of typical syllable transitions that were calculated using SongSeq software according to the method described^8^. SongSeq uses data exported from SAP to identify distinct syllables. Pre-treatment day three was used as the template to compare target datasets generated from recordings made during each treatment day. Distinct syllable types were identified by clustering within a plot of an acoustic feature measure on the y-axis and syllable duration on the x-axis. One or two acoustic features were used as necessary to distinguish two-dimensional syllable clusters. These parameters were optimized for each subject. Once syllable clusters were identified, SongSeq calculated probabilities of each possible pair of syllable types being produced on that recording day. The highest probability transition on the baseline day for each syllable type was designated the “typical transition” for that syllable pair. The percentage of typical transitions for each treatment day was then calculated as the number of typical transitions divided by total transitions x 100%.

## Supplemental statistical analyses details

Potential lesion extent differences across drug treatment groups were assessed using 1-way ANOVA. Lesion extent data are expressed as means +/- SEM. Treatment group differences in microglial migration to lesion sites were determined using a two-tailed t-test. To assess differences in phonology and syllable sequencing over experiment day and across treatment and surgery groups we used a mixed-effects modeling approach with SPSS software (version 26). This method controls for lack of independence of repeated measures derived from single zebra finches (e.g. 17 daily measures from each zebra finch^11^).

For mixed model analysis of phonology and syntax data, individual zebra finches were treated as random subjects and surgery condition (microlesion or sham-microlesion), drug treatment (vehicle, 10 mg/kg CBD [used as a positive control], 3 mg/kg + 0.02, 0.08, 1, 3 and 5% THC), and experiment day were used as fixed factors. For all mixed model analyses, the variance components covariance structure and the maximum likelihood method were used. Fixed explanatory variables were successively added to models. Improvements to simpler models gained by variable additions were determined through likelihood ratio (LR) tests of differences between -2*log likelihood values from the fit of each model to vocal behavior data. For each assessment, models included zebra finch ID as a random factor to control for repeated measures, and surgery condition was added as the first fixed explanatory variable followed by drug treatment and experimental day. Differences between vehicle control and CBD-treated groups were determined following pairwise comparisons using the Bonferroni post-hoc correction. Probabilities less than 0.05 were considered significant. Mixed model statistics reported below are mean differences +/- SEM.

# Results

## Supplemental mixed models analysis details

The mixed model fit of vocal behavior measures to explanatory variables was optimized as described previously^2^. Adding surgery condition as a fixed factor to models with zebra finch ID as a random factor significantly improved their fit to: (1) KL distance measures of phonology: likelihood ratio [LR] = 4255 – 4238 = Χ^2^ 574, 1 d.f., *p* < 0.001) and; (2) percent typical syllable transition measures of syntax (LR = 10456 – 10418 = Χ^2^ 38, 1 d.f., *p* < 0.001). These results demonstrated significant differences across surgery condition for both measures. Next, treatment group (vehicle, 10 mg/kg CBD, 3 mg/kg + 0.02, 0.08, 1, 3 and 5% THC) was added as a fixed factor. This addition further improved model fit to: KL distance data (LR = 4238 – 4225 = Χ^2^ 13, 1 d.f., *p* < 0.001) and; percent typical syllable transition data (LR = 10418 – 10400 = Χ^2^ 18, 2 d.f., *p* < 0.001). This demonstrated significant differences across drug treatment groups for both vocal behavioral measures. Finally, experiment day was added to models as fixed factors that further improved fit of: KL distance data (LR = 4225 – 3160 = Χ^2^ 1065, 1 d.f., *p* < 0.001) and; percent typical syllable transition data (LR = 10400 – 8915 = Χ^2^ 1485, 1 d.f., *p* < 0.001). This demonstrated significant differences across experiment day for both phonology and syntax measures.

# Discussion

## Supplemental discussion of intermediate CBD efficacy

The HVC microlesion pre-clinical model was originally established through a dose-response experiment that investigated effects of 10-fold increasing CBD dosages on vocal recovery^2^. Results indicated approximately threshold responses following 1 mg/kg treatments and full efficacy at 10 mg/kg CBD. The purified botanically derived CBD (≥98%) extract used in this prior work contained the same trace (0.02%) amount of THC as that used presently.

In an effort to produce an intermediate level of response subject to both positive and negative modulation by additional treatments (e.g. increasing THC concentrations) we employed a half-log dosage of 3 mg/kg CBD + 0.02% THC as an intermediate dose between the prior 1 mg/kg threshold and 10 mg/kg full-efficacy dosages. In-terms of phonology, although this 3 mg/kg CBD + 0.02% THC dosage appeared slightly less effective than the 10 mg/kg CBD positive control, the difference was not statistically significant, and the lower dosage was equally effective in reducing KL distance measures on the first two recovery days relative to vehicle controls (Fig 3D). In contrast, on measures of syntax, 3 mg/kg CBD + 0.02% THC was less effective than 10 mg/kg CBD to reduce the acute magnitude of disruption immediately following microlesions, and no more effective than vehicle (Fig 3D, note that this treatment did hasten phonology recovery relative to vehicle).

These results demonstrate a challenge in establishing a CBD treatment with clear intermediate efficacy on vocal recovery. This difficulty is likely due to a combination of modest response levels separating threshold and the fully-effective 10 mg/kg CBD dosages, subject variability inherent to all behavioral experiments and well-established complex phytocannabinoid effects^12^. Clear intermediate efficacy was also difficult to produce due to interesting differential responsiveness of phonology and syntax measures. These sensitivity differences were more evident at the higher THC concentrations as detailed below.

## Supplemental discussion of THC modulation of CBD efficacy

### Phonology

The modestly-improved KL distance measures of phonology produced by 3 mg/kg CBD + 0.02% THC were not observed in zebra finches treated with 3 mg/kg CBD + 0.08% THC (compare Fig 3D and E). This suggests that even small differences in THC content may significantly influence activity of CBD-enriched extracts. It may also be important that the 0.02% and 0.08% THC-containing CBD extracts were from independent lots and so likely differed in content of other trace phytochemicals. Our results add to accumulating evidence suggesting that even small differences in amounts of trace phytocannabinoids can significantly influence extract activity^13,14^.

In contrast to 3 mg/kg CBD + 0.02% THC, the higher THC concentration treatments (3 mg/kg CBD + 0.08, 1, 3 and 5% THC) were all less effective than the 10 mg/kg CBD positive control to reduce KL distance measures. Notably, the highest 3 mg/kg CBD + 5% THC dosage was distinguished not only by lower efficacy than the 10 mg/kg CBD positive control, but also by slower restoration of phonology than the vehicle-treated group. This clearly demonstrates an effect of higher THC concentrations to oppose CBD-promoted recovery of phonology-relevant spectral vocal features. Transforming the 5% THC treatment to dosing units results in 0.15 mg/kg, which approximates the threshold THC dosage for locomotor effects in mice^15^. This raises the possibility that the effect of the 5% THC-containing treatment to delay recovery of phonology may involve motor inhibition. This possibility seems more likely given that vocal recovery following HVC microlesions requires auditory-dependent sensorimotor learning^5^.

### Syntax

In-terms of reducing time to recovery of syntax, each of the 3 mg/kg CBD dosages (+ 0.02, 0.08, 1, 3 and 5% THC) were similar in efficacy to the 10 mg/kg CBD positive control. In addition to decreasing recovery time relative to vehicle controls, 3 mg/kg CBD + 5% THC also reduced the acute magnitude of syntax disruptions in a manner similar to the 10 mg/kg CBD positive control (Fig 4H). This suggests a protective effect of the highest THC concentration that contrasts with its slowing of phonology recovery discussed above. The second highest 3 mg/kg CBD + 3% THC dosage did not produce this acute reduction in magnitude of syntax disruption but did produce a novel effect to more fully restore percent typical transitions to pre-microlesion levels prior to the end of experiments. This was evident from typical syllable transition levels significantly higher than the 10 mg/kg CBD positive control on the last two recovery days (Fig 4G, it may be important that, although there were not significant differences in lesion extent across treatment groups, zebra finches receiving 3 mg/kg CBD + 3% THC did have the lowest mean percent HVC damaged). This ability to more completely recover syntax was not noted in our earlier work. This effect raises the possibility that other zebra finches in the other treatment groups would more completely recover if followed over a period longer than the 10 days used here. Lack of this effect in the higher 3 mg/kg CBD + 5% THC group is consistent with the type of “inverted U-shaped” dose response that is now well-documented for CBD and other phytocannabinoids^12,16^.

## Supplemental discussion of differential efficacy on phonology and syntax

We previously found that purified botanically derived CBD (≥98%) mitigates microlesion effects to disrupt phonology and syntax, and viewed these measures as indicative of a single comprehensive effect: to improve vocal recovery^2^. The present results suggest that this interpretation was too limited, and phonology and syntax are subject to differential phytocannabinoid modulation. By lowering the CBD dosage, and adding THC, phonology and syntax were independently influenced, demonstrating dependence upon distinct underlying processes with individual phytocannabinoid sensitivities. The first step in understanding differential efficacy involves appreciation of the underlying physiological substrates.

### Physiological substrates underlying phonology and syntax

Phonology and syntax are largely controlled by distinct, but converging, neural signaling pathways that interconnect brain regions important to vocal learning and motor production of song (see Fig 6). It may be helpful to consider these pathways as separate systems. For example, the pre-vocal motor brain region we have targeted with microlesions, HVC, is most clearly involved in regulating the temporal structure of song^17,18^. Thus, activity within HVC plays a prominent role in controlling syntax. It is notable that 10 mg/kg CBD is clearly effective in reducing microglial migration to HVC microlesion sites (Fig 5). This suggests a potentially direct anti-inflammatory effect of CBD to stabilize HVC function and related syntax.

Control of the phonological or spectral features of song syllables is more complex and related to combined influences of a learning-essential circuit that includes basal ganglia; the anterior forebrain pathway (AFP, reviewed by Bertram et al., 2014, see Fig 6) and the vocal motor cortical-like region, robust nucleus of the arcopallium (RA, Miller et al., 2017; Sober et al., 2008). Notably, the AFP contributes variability to song structure^20,23^ and its excitatory output is integrated with that of HVC that also projects to and modulates activity of motor cortical RA^24,25^. The vocal variability produced by the AFP is essential to normal vocal learning that requires progressive sensorimotor refinement of vocalizations^23^.

Excitatory efferents from each of these regions (HVC, the AFP and motor cortical RA) are also subject to modulation by populations of inhibitory GABAergic interneurons^26–28^. This is potentially important as this type of inhibitory regulation is of a type known to be produced within mammalian cortex by endocannabinoid depolarization-induced synaptic inhibition (DSI, Wilson et al., 2001). Thus, the activity of these three convergent and interacting systems and their intrinsic regulation likely all contribute to the ability of phytocannabinoid treatments to influence learning-dependent vocal recovery.

### Factors influencing vocal recovery

Microlesion recovery requires sensorimotor learning that depends upon auditory feedback and does not occur in deafened zebra finches^5^. Similar, but more slowly manifested vocal deficits occur in zebra finches unable to appropriately modify vocal output due either to deafening, or denervation of the vocal organ^30,31^. These vocal deficits following lack of auditory feedback also depend upon AFP output to vocal motor cortex, further demonstrating a key role in maintenance and recovery of learned vocalizations^32^. It is also worth noting that the vocal disrupting effects of HVC microlesions are not apparent in zebra finches with prior bilateral AFP lesions^33^ – indicating that this vocal learning circuit is essential to modification of already learned song, and that its loss effectively crystalizes the behavior.

Importantly, the auditory input that is critical to vocal recovery depends upon midbrain dopaminergic signaling^34^. Accumulating evidence indicates that zebra finch vocal learning (and therefore the learning-dependent vocal recovery studied here) involves motivated behavior reinforced by dopaminergic transmission^35^. This dopaminergic activity shares features across vertebrates and involves projections from ventral tegmentum that release dopamine into regions of striatum and pallidum^36^. In zebra finches, this signaling functions as a learning-related predictor of performance errors^37,38^. A remarkably similar signaling system controls mammalian perception of outcome-predictive cues^39^. This is relevant to the present study as the mammalian reward-perception system requires endocannabinoid signaling and is inhibited by CB_1_ antagonism^40^. Given other dopaminergic signaling similarities, it is likely that the zebra finch vocal learning-related error detection system is also endocannabinoid dependent, and this is a possibility that should be tested. If confirmed, this represents another substrate liable to modulation by the phytocannabinoid treatments we have employed, particularly given evidence for CBD antagonism of CB_1_ receptor-related signaling (reviewed by McPartland et al., 2015; Pertwee, 2008).

### Systems Implications

Interaction with some or all of these processes likely underlies phytocannabinoid effects on vocal recovery. Microlesions are thought to disrupt the balance between the error-generating AFP and stereotypy-promoting HVC and their dual control over motor cortical RA^19^. Phytocannabinoid effects to improve vocal recovery must in some manner promote or stabilize this balance. Post-microlesion balance of motor cortical input could be promoted, potentially rapidly, by inhibition of AFP afferents (perhaps via activation of inhibitory interneurons), augmentation of remaining intact HVC projections, or to enhancement of some balancing regulatory process present and active within RA itself. CBD clearly has rapid anti-inflammatory activity in this system, as demonstrated by reduced microglial densities near microlesion sites (Fig 5). It will be important to investigate potential distal effects within HVC’s efferent targets that include the AFP and motor cortical RA (Fig 6).

In addition to rapid effects to reduce the acute magnitude of microlesion effects, CBD promoted the more gradual processes of recovery of phonology and syntax to pre-microlesion levels. These slower processes likely involve the auditory-dependent sensorimotor feedback that is both required for recovery, and mediated by midbrain-striatal dopaminergic signaling. In the cases where treatments resulted in reduced time to recovery, this may have involved promotion of dopamine signaling that drives motivated vocal learning. In the case of slowed recovery of phonology in zebra finches treated with 3 mg/kg CBD + 5% THC, interference with this reinforcement-related process may be involved. Notably, this is the only clear case observed to date of a CBD treatment slowing recovery in the HVC microlesion model (Fig 3H). The simplest explanation for this involves increased CB_1_-related agonism by THC consistent with effects of the full agonist WIN55212-2 to disrupt vocal learning developmentally^43,44^. This is a possibility to consider for future experiments, and could be tested by evaluating ability of the CB_1_-selective antagonist SR141716A to restore speed of vocal recovery. It may also be the case that higher concentrations of THC effectively oppose CBD effects to improve recovery. This would be consistent with a complicated literature supporting both additive and antagonistic effects of combined CBD and THC treatments^12^. Although 3 mg/kg CBD + 5% THC clearly slowed recovery of phonology, pre-microlesion levels were restored prior to completion of experiments, and so the treatment was not itself disruptive *per se*. Also, the acoustic variability associated with AFP input to vocal motor cortex is essential to the normal process of vocal learning^45^. And so, it may be too narrow to consider this delay as a deficit in recovery – particularly as it reduced the magnitude of microlesion effects upon syntax (Fig 4H), and the 3 mg/kg CBD + 3% THC treatment improved time to syntax recovery (Fig 4G).

# Supplementary conclusions

The complex efficacy and responsiveness that we observed was likely a function of the distinct physiology and sensitivities of underlying neural substrates. Identifying distinct effects within these interconnected cortical, striatal and thalamic circuits is our focus going forward. Given relatively few vocal learning species (humans are the sole primate example^46^) and similarities of brain regions and circuits involved^47^, what we find by studying phytocannabinoid effects on zebra finch vocal recovery promises to reveal new therapeutic mechanisms and applications.

# References

1. Schluttenhofer C and Yuan L. Challenges towards Revitalizing Hemp: A Multifaceted Crop. Trends in Plant Science 2017;22(11):917–929; doi: 10.1016/j.tplants.2017.08.004.

2. Alalawi A, Dodu JC, Woolley-Roberts M, et al. Cannabidiol Improves Vocal Learning-Dependent Recovery from, and Reduces Magnitude of Deficits Following, Damage to a Cortical-like Brain Region in a Songbird Pre-Clinical Animal Model. Neuropharmacology 2019;158:107716; doi: 10.1016/j.neuropharm.2019.107716.

3. Gamble L-J, Boesch JM, Frye CW, et al. Pharmacokinetics, Safety, and Clinical Efficacy of Cannabidiol Treatment in Osteoarthritic Dogs. Front Vet Sci 2018;5; doi: 10.3389/fvets.2018.00165.

4. Tchernichovski O, Nottebohm F, Ho CE, et al. A Procedure for an Automated Measurement of Song Similarity. Anim Behav 2000;59(6):1167–1176; doi: 10.1006/anbe.1999.1416.

5. Thompson JA, Wu W, Bertram R, et al. Auditory-Dependent Vocal Recovery in Adult Male Zebra Finches Is Facilitated by Lesion of a Forebrain Pathway That Includes the Basal Ganglia. J Neurosci 2007;27(45):12308–12320; doi: 10.1523/JNEUROSCI.2853-07.2007.

6. Nixdorf-Bergweiler BE, Bischof H-J, Nixdorf-Bergweiler BE, et al. A Stereotaxic Atlas Of The Brain Of The Zebra Finch, Taeniopygia Guttata. National Center for Biotechnology Information (US); 2007.

7. Wu W, Thompson JA, Bertram R, et al. A Statistical Method for Quantifying Songbird Phonology and Syntax. Journal of Neuroscience Methods 2008;174(1):147–154; doi: 10.1016/j.jneumeth.2008.06.033.

8. Daou A, Johnson F, Wu W, et al. A Computational Tool for Automated Large-Scale Analysis and Measurement of Bird-Song Syntax. Journal of Neuroscience Methods 2012;210(2):147–160; doi: 10.1016/j.jneumeth.2012.07.020.

9. Soderstrom K and Alalawi A. Software for Objective Comparison of Vocal Acoustic Features over Weeks of Audio Recording: KLFromRecordingDays. SoftwareX 2017;6:271–277; doi: 10.1016/j.softx.2017.10.003.

10. Grubbs FE. Procedures for Detecting Outlying Observations in Samples. Technometrics 1969;11(1):1; doi: 10.2307/1266761.

11. Aarts E, Verhage M, Veenvliet JV, et al. A Solution to Dependency: Using Multilevel Analysis to Accommodate Nested Data. Nat Neurosci 2014;17(4):491–496; doi: 10.1038/nn.3648.

12. Zuardi AW, Hallak JEC and Crippa JAS. Interaction between Cannabidiol (CBD) and Δ9- Tetrahydrocannabinol (THC): Influence of Administration Interval and Dose Ratio between the Cannabinoids. Psychopharmacology 2012;219(1):247–249; doi: 10.1007/s00213-011-2495-x.

13. Berman P, Futoran K, Lewitus GM, et al. A New ESI-LC/MS Approach for Comprehensive Metabolic Profiling of Phytocannabinoids in Cannabis. Scientific Reports 2018;8(1):1–15; doi: 10.1038/s41598-018-32651-4.

14. Namdar D, Voet H, Ajjampura V, et al. Terpenoids and Phytocannabinoids Co-Produced in Cannabis Sativa Strains Show Specific Interaction for Cell Cytotoxic Activity. Molecules 2019;24(17):3031; doi: 10.3390/molecules24173031.

15. Varvel SA, Wiley JL, Yang R, et al. Interactions between THC and Cannabidiol in Mouse Models of Cannabinoid Activity. Psychopharmacology 2006;186(2):226–234; doi: 10.1007/s00213-006-0356-9.

16. Zuardi AW, Rodrigues NP, Silva AL, et al. Inverted U-Shaped Dose-Response Curve of the Anxiolytic Effect of Cannabidiol during Public Speaking in Real Life. Front Pharmacol 2017;8:259; doi: 10.3389/fphar.2017.00259.

17. Hahnloser RHR, Kozhevnikov AA and Fee MS. An Ultra-Sparse Code Underlies the Generation of Neural Sequences in a Songbird. Nature 2002;419(6902):65–70; doi: 10.1038/nature00974.

18. Long MA and Fee MS. Using Temperature to Analyse Temporal Dynamics in the Songbird Motor Pathway. Nature 2008;456(7219):189–194; doi: 10.1038/nature07448.

19. Bertram R, Daou A, Hyson RL, et al. Two Neural Streams, One Voice: Pathways for Theme and Variation in the Songbird Brain. Neuroscience 2014;277:806–817; doi: 10.1016/j.neuroscience.2014.07.061.

20. Kao MH and Brainard MS. Lesions of an Avian Basal Ganglia Circuit Prevent Context-Dependent Changes to Song Variability. Journal of Neurophysiology 2006;96(3):1441–1455; doi: 10.1152/jn.01138.2005.

21. Miller MN, Cheung CYJ and Brainard MS. Vocal Learning Promotes Patterned Inhibitory Connectivity. Nature Communications 2017;8(1):1–9; doi: 10.1038/s41467-017-01914-5.

22. Sober SJ, Wohlgemuth MJ and Brainard MS. Central Contributions to Acoustic Variation in Birdsong. J Neurosci 2008;28(41):10370–10379; doi: 10.1523/JNEUROSCI.2448-08.2008.

23. Olveczky BP, Andalman AS and Fee MS. Vocal Experimentation in the Juvenile Songbird Requires a Basal Ganglia Circuit. PLoS Biol 2005;3(5):e153; doi: 10.1371/journal.pbio.0030153.

24. Herrmann K and Arnold AP. The Development of Afferent Projections to the Robust Archistriatal Nucleus in Male Zebra Finches: A Quantitative Electron Microscopic Study. J Neurosci 1991;11(7):2063–2074.

25. Kittelberger JM and Mooney R. Lesions of an Avian Forebrain Nucleus That Disrupt Song Development Alter Synaptic Connectivity and Transmission in the Vocal Premotor Pathway. J Neurosci 1999;19(21):9385–9398.

26. Farries MA, Ding L and Perkel DJ. Evidence for “Direct” and “Indirect” Pathways through the Song System Basal Ganglia. Journal of Comparative Neurology 2005;484(1):93–104; doi: 10.1002/cne.20464.

27. Scotto-Lomassese S, Rochefort C, Nshdejan A, et al. HVC Interneurons Are Not Renewed in Adult Male Zebra Finches. Eur J Neurosci 2007;25(6):1663–1668; doi: 10.1111/j.1460-9568.2007.05418.x.

28. Spiro JE, Dalva MB and Mooney R. Long-Range Inhibition within the Zebra Finch Song Nucleus RA Can Coordinate the Firing of Multiple Projection Neurons. J Neurophysiol 1999;81(6):3007–3020; doi: 10.1152/jn.1999.81.6.3007.

29. Wilson RI, Kunos G and Nicoll RA. Presynaptic Specificity of Endocannabinoid Signaling in the Hippocampus. Neuron 2001;31(3):453–462; doi: 10.1016/S0896-6273(01)00372-5.

30. Nordeen KW and Nordeen EJ. Auditory Feedback Is Necessary for the Maintenance of Stereotyped Song in Adult Zebra Finches. Behav Neural Biol 1992;57(1):58–66.

31. Williams H and McKibben JR. Changes in Stereotyped Central Motor Patterns Controlling Vocalization Are Induced by Peripheral Nerve Injury. Behav Neural Biol 1992;57(1):67–78; doi: 10.1016/0163-1047(92)90768-y.

32. Nordeen KW and Nordeen EJ. Deafening-Induced Vocal Deterioration in Adult Songbirds Is Reversed by Disrupting a Basal Ganglia-Forebrain Circuit. J Neurosci 2010;30(21):7392–7400; doi: 10.1523/JNEUROSCI.6181-09.2010.

33. Thompson JA and Johnson F. HVC Microlesions Do Not Destabilize the Vocal Patterns of Adult Male Zebra Finches with Prior Ablation of LMAN. Devel Neurobio 2007;67(2):205–218; doi: 10.1002/dneu.20287.

34. Gale SD, Person AL and Perkel DJ. A Novel Basal Ganglia Pathway Forms a Loop Linking a Vocal Learning Circuit with Its Dopaminergic Input. J Comp Neurol 2008;508(5):824–839; doi: 10.1002/cne.21700.

35. Hisey E, Kearney MG and Mooney R. A Common Neural Circuit Mechanism for Internally Guided and Externally Reinforced Forms of Motor Learning. Nat Neurosci 2018;21(4):589–597; doi: 10.1038/s41593-018-0092-6.

36. Gale SD and Perkel DJ. Anatomy of a Songbird Basal Ganglia Circuit Essential for Vocal Learning and Plasticity. J Chem Neuroanat 2010;39(2):124; doi: 10.1016/j.jchemneu.2009.07.003.

37. Chen R, Puzerey PA, Roeser AC, et al. Songbird Ventral Pallidum Sends Diverse Performance Error Signals to Dopaminergic Midbrain. Neuron 2019;103(2):266-276.e4; doi: 10.1016/j.neuron.2019.04.038.

38. Gadagkar V, Puzerey PA, Chen R, et al. Dopamine Neurons Encode Performance Error in Singing Birds. Science 2016;354(6317):1278–1282; doi: 10.1126/science.aah6837.

39. Wenzel JM and Cheer JF. Endocannabinoid-Dependent Modulation of Phasic Dopamine Signaling Encodes External and Internal Reward-Predictive Cues. Front Psychiatry 2014;5:118; doi: 10.3389/fpsyt.2014.00118.

40. Cheer JF, Wassum KM, Sombers LA, et al. Phasic Dopamine Release Evoked by Abused Substances Requires Cannabinoid Receptor Activation. J Neurosci 2007;27(4):791–795; doi: 10.1523/JNEUROSCI.4152-06.2007.

41. McPartland JM, Duncan M, Di Marzo V, et al. Are Cannabidiol and Δ(9) -Tetrahydrocannabivarin Negative Modulators of the Endocannabinoid System? A Systematic Review. Br J Pharmacol 2015;172(3):737–753; doi: 10.1111/bph.12944.

42. Pertwee RG. The Diverse CB1 and CB2 Receptor Pharmacology of Three Plant Cannabinoids: Delta9-Tetrahydrocannabinol, Cannabidiol and Delta9-Tetrahydrocannabivarin. Br J Pharmacol 2008;153(2):199–215; doi: 10.1038/sj.bjp.0707442.

43. Soderstrom K and Johnson F. Cannabinoid Exposure Alters Learning of Zebra Finch Vocal Patterns. Developmental Brain Research 2003;142(2):215–217; doi: 10.1016/S0165-3806(03)00061-0.

44. Soderstrom K and Tian Q. Distinct Periods of Cannabinoid Sensitivity during Zebra Finch Vocal Development. Brain Res Dev Brain Res 2004;153(2):225–232; doi: 10.1016/j.devbrainres.2004.09.002.

45. Bottjer SW, Miesner EA and Arnold AP. Forebrain Lesions Disrupt Development but Not Maintenance of Song in Passerine Birds. Science 1984;224(4651):901–903.

46. Jarvis ED. Evolution of Vocal Learning and Spoken Language. Science 2019;366(6461):50–54; doi: 10.1126/science.aax0287.

47. Chakraborty M and Jarvis ED. Brain Evolution by Brain Pathway Duplication. Philos Trans R Soc Lond B Biol Sci 2015;370(1684):20150056; doi: 10.1098/rstb.2015.0056.
